# In Vitro Antibacterial Activity of Selected Medicinal Plants in the Traditional Treatment of Skin and Wound Infections in Eastern Ethiopia

**DOI:** 10.1155/2018/1862401

**Published:** 2018-07-11

**Authors:** Bahar Mummed, Ashebr Abraha, Teka Feyera, Adugna Nigusse, Solomon Assefa

**Affiliations:** ^1^Department of Microbiology and Veterinary Public Health, College of Veterinary Medicine, Jigjiga University, Ethiopia; ^2^College of Veterinary Medicine, Haramaya University, Ethiopia; ^3^Department of Veterinary Clinical Studies, College of Veterinary Medicine, Jigjiga University, Ethiopia; ^4^Department of Biomedical Sciences, College of Medicine and Health Sciences, Jigjiga University, Ethiopia; ^5^Department of Pharmacology and Clinical Pharmacy, School of Pharmacy, Addis Ababa University, Ethiopia

## Abstract

**Background:**

External infections involving the skin and wound are the most frequent complications affecting humans and animals. Medicinal plants play great roles in the treatment of skin and wound infections. This study was aimed to evaluate the* in vitro* antibacterial activity of crude methanolic extracts of nine medicinal plants.

**Methods:**

Agar well diffusion and broth dilution methods were used to determine the antibacterial activity of nine Ethiopian plants against four bacterial species including* Staphylococcus aureus*,* Pseudomonas aeruginosa*,* Escherichia coli*, and* Klebsiella pneumoniae*.

**Results:**

Among the tested plants, seven (*Cissus quadrangularis*,* Commelina benghalensis*,* Euphorbia heterophylla*,* Euphorbia prostrate*,* Momordica schimperiana*,* Trianthema *spp., and* Solanum incanum*) were found to exhibit considerable antibacterial activity against at least one of the test bacteria. The extracts of* C. quadrangularis*,* E. heterophylla*, and* E. prostrata* had a wide spectrum of antibacterial activities against test bacterial strains while the extracts of* Grewia villosa* and* Schinus molle *did not show any inhibitory activity. Clinical isolate and laboratory strain of S.* aureus* showed the highest susceptibility to highest concentration (780 mg/mL) of* E. prostrata* with a zone of inhibition of 21.0mm and 22.3mm, respectively.

**Conclusion:**

This study indicates clear evidence supporting the traditional use of seven plants in treating skin and wound infections related to bacteria.

## 1. Background

The skin, being the outermost and first line of defense, is easily exposed to physical agents and different pathogens leading to various infections and wounds [1].Wound, which is a breakage of the skin, results in the loss of continuity of epithelium with or without the loss of underlying connective tissue. Physical, chemical, thermal, microbial, and immunological factors may be responsible for causing wounds in human and animals [2, 3].

Skin infections and topical wounds require special attention as they make human and animal prone to bacterial, fungal, and viral contaminations, thereby making them further susceptible to other types of secondary complications [4]. The most common pathogens isolated from wounds are* Streptococcus *spp.,* Staphylococcus aureus*,* Escherichia coli*,* Pseudomonas aeruginosa*,* Proteus *spp.,* Klebsiella*,* Enterobacter*,* Enterococci*,* Bacteroides*,* Clostridium*,* Candida*,* Peptostreptococcus*,* Fusobacterium,* and* Aeromonas *[5]. These pathogens can seriously delay wound healing process by disrupting the normal clotting mechanisms and promoting disordered leukocyte function and poor quality granulation tissue formation, reduce tensile strength of connective tissue, and impair epithelization [6].

Medicinal plants are effective in the treatment of infectious diseases and infections of various types of external wounds (chronic, deep suppurative, open, lacerated, incised, and ulcerated) and have been used for these purposes in humans and different species of animals [7]. The use of medicinal plants has the added benefit of reducing many of the side effects often associated with synthetic antimicrobials [8, 9].

In Ethiopian Somali region (eastern Ethiopia), various researchers [10–12] have reported ranges of medicinal plants used against different ailments by traditional healers. The majority of the plants are very popular and known to be utilized and even marketed throughout the region for the management of skin and open wound infections. However, limited experimental evidence is available regarding the antibacterial activity of commonly used plant preparations against common bacterial pathogens involved in skin and wound infections. Hence, this study was aimed to investigate the* in vitro* antibacterial activity of crude methanolic extracts of selected medicinal plants against common bacteria involved in skin and wound infections.

## 2. Methods

### 2.1. Collection and Identification of the Plant Materials

Field survey was conducted in Jigjiga woreda, Ethiopian Somali regional state, to identify and collect the potential medicinal plants traditionally used to treat skin and wound infections. Accordingly, nine species of medicinal plants (relevant plant parts) claimed to be traditionally used against skin and wound infections were collected from their natural habitat (9°20′60.00′′N 42°47′60.00′′E) with the guide of traditional healers. The collected plants were* Cissus quadrangularis* (aerial),* Commelina benghalensis *(leaves),* Euphorbia heterophylla *(root),* Euphorbia prostrate *(whole),* Grewia villosa *(leaves),* Momordica schimperiana *(fruit),* Trianthema *spp. (aerial),* Schinus molle *(leaves), and* Solanum incanum *(fruit).

The collected plant specimens were identified using herbarium materials and taxonomic keys described in various volumes on the Flora of Ethiopia [13]. Voucher specimens were deposited in the Herbarium of the Department of Plant Science, Haramaya University, Ethiopia.

### 2.2. Crude Extract Preparation

Shade-dried and coarsely powdered plant materials were subjected to extraction using absolute methanol by maceration technique. A total of 250g of each powdered materials were separately mixed with the extraction solvent (100 g of powder in 1000 ml of solvent proportion) in Erlenmeyer flasks. The flasks were left on a mechanical shaker at 150 rpm for 24 hr at room temperature for three days and then filtered through Whatman No. 1 filter paper using Buchner funnel. The procedure was repeated three times on the marc to allow the solvent extract substantial quantities of the chemical constituents from the pounded plant materials. The extracts were further concentrated to dryness under reduced pressure at 37°C using a Buchi rotary evaporator. The yields from the different extracts were weighed and recorded and the resulting extracts were then transferred into well labeled vials and kept at 4°C until required for use. Sterility of filtered extracts was checked by plating them on Muller-Hinton agar (Bacton Dickinson and Company, Cockeysville, MD, USA) [14].

### 2.3. Phytochemical Screening

Crude extracts of each plant were screened for the presence and absence of different phytochemical constituents to relate the secondary metabolites with antibacterial activity. Hence, tests for alkaloids, flavonoids, glycosides, phenolic compounds, saponins, sterols, and tannins were carried out following standard procedures described by Trease and Evans (1989) [15] and Sofowora (1993) [16].

### 2.4. Sources of Test Organisms

The test organisms were clinical isolates and laboratory strains of four bacteria species, namely,* Staphylococcus aureus*,* Pseudomonas aeruginosa*,* Escherichia coli,* and* Klebsiella pneumoniae*. These bacteria were selected based on their potential to cause skin and wound infections.

#### 2.4.1. Clinical Sample Collection and Test Bacterial Isolation

The samples were collected from various animal species (cattle, goat, sheep, donkey, and camel) coming to Jigjiga town Veterinary Clinic for open wound management. Before sample collection, the animals were restrained and the skin around the lesion was disinfected using cotton wool soaked in 70% alcohol to avoid any extraneous contamination. A total of 23 swab samples were aseptically collected using sterile cotton swabs and immediately immersed into peptone water and transported using a box containing an ice to the microbiology laboratory at Jigjiga University, College of Veterinary Medicine.

The collected swabs were streaked on plates of blood agar, eosin methylene blue agar, MacConkey agar, nutrient agar, and mannitol salt agar by sterile inoculation loop. The plates were incubated at 37°C for 24–48 hr. After incubation, cultures were examined for significant growth [17]. Subcultures were made onto plates of nutrient agar and incubated for another 24hr.

#### 2.4.2. The Standard Laboratory Strains

The standard laboratory strains of* S. aureus* [American Type Culture Collection (ATCC) 25923],* P. aeruginosa *(ATCC 27853),* E. coli* (ATCC 25922), and* K.pneumoniae* (ATCC 700603) were obtained from the Ethiopia Public Health Institute (EPHI). Purity and viability of the organisms were checked by plating, gram staining, and conducting primary and secondary biochemical tests.

#### 2.4.3. Inoculums Preparation and Standardization

The standard and clinical isolates were inoculated and spread on prepared agar plates using inoculating wire loop following aseptic condition and incubated for 24 hr at 37°C. Then, the bacterial turbidity of each species was prepared and standardized by following the guideline of Clinical and Laboratory Standard Institute (CLSI) [18]. The test suspension was standardized to match 0.5 McFarland turbidity standard which corresponds to approximately 1 × 10^8^ CFU/mL.

### 2.5. Antibacterial Activity Test of Individual and Combined Crude Extracts

To evaluate the antibacterial activity of the individual crude extracts and their combination, the antibacterial agar well diffusion assay was employed following the methods described by different works [19–21]. The standardized bacterial broth culture was streaked evenly on sterile Muller-Hinton agar (MHA) plates with a cotton swab. One liter of MHA contains beef infusion (2.0 g), acid hydrolysate of casein (17.5g), starch (1.5g), and agar (17.0g) with a final pH of 7.3±0.2. After thirty minutes, on each plate, equidistant wells were made with a 6mm diameter sterilized cork borer. The labeled wells were filled with 100*μ*L of 780, 390, and 195 mg/mL of test extracts. For comparison, gentamicin (25 *μ*g/mL) and sterile distilled water (100 *μ*L/well) were used as a positive and negative control, respectively. Then, the plates were allowed to stand on the laboratory bench for 2 hr to allow proper diffusion of the extracts into the media. Finally, the plates were incubated at 37°C for 24 hr. After incubation, the resulting diameters of zones of inhibition, including the diameter of the well, were measured using a ruler and reported in millimeter (mm). For each bacterium, the experiment was performed in three independent tests and the mean of zones of inhibition was calculated for each test extract and the standard antibiotic.

After running the agar well diffusion assay for the individual extracts, crude extracts of three medicinal plants which exhibited relatively higher* in vitro* antibacterial efficacy were combined in a proportion of 1:1 and 1:1:1 to form a combination of two and three, respectively. The combined extracts were then subjected to a similar antibacterial activity test using agar well diffusion as described above.

#### 2.5.1. Determination of Minimum Inhibitory Concentration

The minimum inhibitory concentration (MIC) of individual and combined plant extracts was determined by broth dilution method according to the method described by Chung et al. [22]. To determine the MICs, the extracts were dissolved in methanol to give a stock concentration of 10240 *μ*g/mL while the antibiotics were dissolved in ultrapure water to give stock concentrations of 5120 *μ*g/mL. All stock concentrations of compounds and antibiotics were filter-sterilized using 0.20 *μ*m syringe filter. MIC was determined for extracts that showed growth inhibition diameter of ≥8mm at 780 mg/ml concentration. Twofold serial dilutions of the extracts were made with nutrient broth. Extract solution of 390 mg/mL were serially diluted in ten test tubes to the concentrations of 390, 195, 97.5, 48.75, 24.38, 12.18, 6.09, 3.05, 1.52, and 0.76 mg/mL. Microbial suspension of 1 mL was added to each of the tubes and incubated at 37°C for 24 hr. The control tubes did not have test extract but contained the test bacteria and the sterile distilled water used to dissolve the extracts. After incubation, the visual turbidity was observed and recorded. The lowest concentration in which the turbidity was not observed was measured as a MIC of the individual or combined of extracts.

### 2.6. Data Analysis

Data obtained from the experiment were analyzed using SPSS, version 20. The statistical differences of the mean zone of inhibition of extract for individual bacterium were carried out by employing ANOVA followed by Tukey's Post Hoc Multiple Comparison test at a significance level of P<0.05. The MIC was analyzed using descriptive statistics.

## 3. Results

### 3.1. Phytochemical Screening

According to the qualitative phytochemical screening, the maximum bioactive secondary metabolites were found in* E. heterophylla* and* E. prostrate *(Table 1).

### 3.2. Antibacterial Activity

#### 3.2.1. The Agar Well Diffusion Assay

The agar well diffusion assay revealed that seven of the nine evaluated medicinal plants were found to exhibit a considerable antibacterial activity against at least one of the test bacteria. Susceptibility of the four tested bacteria to the extracts varied with considerable discrepancies between the clinical isolates and standard strains. Extracts with colony growth inhibitory effect at the highest dose showed a mean zone of inhibition ranged from 8.7 to 22.3mm. Gentamicin showed a significant superiority (p<0.05) in the zone of inhibition as compared to the test extracts (Table 2).

For most of the test extracts, the highest concentration (780 mg/mL) exhibited a significantly higher (P<0.05) zone of inhibition as compared to the respective lowest concentration (195 mg/mL). The strongest antibacterial activity with maximum zone of inhibition (22.3mm) was recorded with methanolic extract of* E. prostrate *against standard strain of* S. aureus *at 780mg/ml of concentration (Table 2).

In the present study, three plant extracts with a better inhibition activity (*C. quadrangularis*,* E. heterophylla*, and* E. prostrata*) were selected to evaluate their combination effect against test bacteria at concentration of 780 mg/mL, 390 mg/mL, and 195 mg/mL. Accordingly, the antibacterial activity was evaluated for the combinations of* C. quadrangularis *and* E. heterophylla *(CqEh)*, C. quadrangularis* and* E. prostrata* (CqEp),* E. heterophylla *and* E. prostrata* (EhEp), and* C. quadrangularis, E. heterophylla *and* E. prostrata* (CqEhEp) extracts in a proportion of 1:1 and 1:1:1 combination (Table 3). The combination of the three plant extracts at highest dose (780 mg/ml) showed a comparable zone of inhibition (P<0.05) with the standard drug against clinical and standard strain of* S. aureus* and clinical isolate of* P. aeruginosa.*

#### 3.2.2. MIC of Extracts

The MIC values of active plant extracts ranged from12.18 mg/mL to 390 mg/mL (Table 4). Of all the crude extracts evaluated,* E. prostrata* had considerable antibacterial activity with MIC value of 12.18 mg/mL against clinical isolates and standard strains of* S. aureus*.

The MIC of most of the combined extracts was lower compared to the MIC of individual extracts. EhEp and CqEhEp combinations exhibited the least average MIC value of 2.03 mg/ml against* S. aureus *(ATCC25923) (Table 5).

## 4. Discussion

Due to a high incidence of antibiotic resistance, evaluating the antibacterial effect of herbal medicines as potent agents of treating skin and wound infections has a paramount importance in addressing animal as well as human health problems [23, 24]. In the present study, antibacterial activity evaluation was performed for individual and combined crude plant extracts against Gram-positive and Gram-negative bacteria.

The highest activity was recorded with the crude extract of* E. prostrata* at 780 mg/mL concentration followed by the extracts of* E. heterophylla *and C*. benghalensis* against a standard strain of* S. aureus. *Previous studies [25, 26] showed a similar activity on ethanol leaves extract and solvent fractions of* E. heterophylla *against* S. aureus*,* P. aeruginosa*,* E. coli, Streptococcus pneumonia*, and* K. pneumoniae.*

The extract of* C. quadrangularis *showed highest inhibitory activity (18.3mm) against clinical and standard strains of S*. aureus. *The current observation on the inhibitory activity of* C. quadrangularis *against* S. aureus *was more pronounced than a study done by Mengiste et al. [27].

This study also indicated that combinations of the extracts showed enhancement of the activity of less active plants in individual extract through decreasing the MIC value. Separately administered* C. quadrangularis*,* E. heterophylla*, and* E. prostrate *showed less antibacterial activity against* P. aeruginosa*. However, the combinations of the plant extracts of CqEh, CqEp, EhEp, and CqEhEp showed improved antibacterial activity against* P. aeruginosa*. Generally, the combinations of extracts can lead to additive or synergistic effects [28, 29]. Probably, the main reasons for this are sequential inhibition of a common biochemical pathway and disintegration of the outer membrane [30, 31]. Since the majority of the combination exhibited MIC value of below 8mg/mL, they have noteworthy antimicrobial activity [32].

MIC assay was employed to evaluate the effectiveness of the extracts to inhibit the growth of the tested bacteria. The plant extracts with high activity against a particular organism usually give low MIC value while the extracts with low activity give high MIC value [33]. In consonance with this general assertion, in the present study, the MIC value of the extracts agreed with their corresponding antibacterial activities. The MIC value of* E. prostrata* ranged from 12.18 mg/mL to 390.00 mg/mL against* S. aureus* (clinical and standard) and* E. coli* (clinical), respectively.

The antibacterial properties of the active plants may be due to the presence of different bioactive chemical agents in the extracts, which are known to act by a different mechanism to exert an antibacterial action. In the present study, medicinal plants containing tannins showed a better antibacterial activity. Mode of action of tannins may be related to their ability to inactivate several enzymes, microbial adhesion, and cell envelope transport proteins [34]. Flavonoids and saponins have been reported to possess antibacterial activity, which could be attributed to their ability to form complex with extracellular proteins, soluble proteins, and bacterial cell wall [35–37].

## 5. Conclusion

All the plant species evaluated in this study are currently used traditionally for the treatment of skin and wound infections. The positive findings from this study provide a scientific basis for the traditional use of* C. quadrangularis*,* C. benghalensis*,* E. heterophylla*,* E. prostrata*,* M. schimperiana*,* Trianthema *spp., and* S. incanum* for treatment of skin and wound infections. The extracts of* C. quadrangularis*,* E. heterophylla*, and* E. prostrata* have a promising antibacterial activity individually and in combination against tested bacteria. Finally, the results of this study clearly elucidate the antibacterial potential of these plants and provide an evidence to support their use in folk medicine.

## Figures and Tables

**Table 1:** Preliminary phytochemical screening of crude extract of the studied plants.

**Plant species**	**Phytochemicals**						
	Alkaloids	Saponins	Tannins	Flavonoids	Glycosides	Sterols	Phenolics
*C. quadrangularis*	++	+	++	++	-	-	+
*C. benghalensis*	+	-	+	++	-	-	+
*E. heterophylla*	++	+	++	+++	+	-	+
*E. prostrata*	+	+	++	++	+	-	+
*G. villosa*	+	-	-	+	-	-	-
*M. schimperiana*	++	+	-	+	-	-	+
*Trianthema* spp.	+	+	+	-	+	-	-
*S. molle*	+	-	-	-	-	-	-
*S. incanum*	+	+	+	+	-	-	+

+++: strongly detected; ++: moderately detected; +: slightly detected; -: not detectable.

**Table 2:** Mean zone of inhibition (mm) of different bacteria at various concentrations of the test extracts of the experimental plants.

Plants	Concentration	Bacteria							
		*S. aureus*		*P. aeruginosa*		*E. coli*		*K. pneumoniae*	
	[mg/ml]								
		Cli	Sta	Cli	Sta	Cli	Sta	Cli	Sta
*C. quadrangularis*	195	10.3±1.8_ _^acd^	10.7±0.7_ _^ad^	10.0±0.6_ _^ad^	10.7±0.3_ _^acd^	- - -	- - -	7.3±0.3_ _^ad^	8.7±0.9_ _^ad^
	390	14.7±0.9_ _^ab^	15.0±1.2_ _^a^	12.0±0.6_ _^a^	14.3±0.7_ _^abd^	7.3±0.3_ _^a^	7.3±0.3_ _^a^	9.3±1.2_ _^ad^	11.7±0.9_ _^ad^
	780	18.3±1.5_ _^ab^	18.3±0.9_ _^ab^	15.7±0.7_ _^ab^	17.7±1.2_ _^abc^	9.3±0.3_ _^ab^	9.7±0.7_ _^ab^	13.3±1.5_ _^abc^	16.0±1.2_ _^abc^

*C. benghalensis*	195	9.3±1.5_ _^ad^	13.7±0.9_ _^ad^	- - -	8.0±0.6_ _^ad^	- - -	- - -	- - -	- - -
	390	13.0±1.2_ _^a^	17.0±0.6_ _^a^	7.3±0.3_ _^a^	11.0±0.6_ _^ad^	- - -	- - -	- - -	- - -
	780	15.7±1.8_ _^ab^	20.0±1.5_ _^ab^	10.7±0.3_ _^ab^	14.7±0.7_ _^abc^	- - -	- - -	- - -	- - -

*E. heterophylla*	195	13.3±0.3_ _^ad^	14.0±0.0_ _^ad^	8.0±0.6_ _^ad^	10.3±0.9_ _^ad^	8.3±0.7_ _^ad^	10.7±0.3_ _^ad^	8.7±0.3_ _^acd^	13.0±0.0_ _^ad^
	390	14.7±0.9_ _^a^	16.7±0.9_ _^a^	11.7±0.9_ _^a^	13.0±0.6_ _^a^	11.0±0.6_ _^ad^	13.3±1.2_ _^a^	12.0±0.6_ _^abd^	14.7±0.9_ _^a^
	780	18.7±1.2_ _^ab^	20.3±0.0_ _^ab^	14.3±1.5_ _^ab^	15.3±0.9_ _^ab^	14.0±0.0_ _^abc^	16.0±1.7_ _^ab^	15.7±0.3_ _^abc^	18.0±0.0_ _^ab^

*E. prostrata*	195	14.3±0.3_ _^ad^	15.7±0.3_ _^ad^	9.7±0.7_ _^ad^	11.3±0.7_ _^ad^	- - -	7.3±0.6_ _^ad^	8.0±0.6_ _^ad^	11.3±0.9_ _^ad^
	390	17.7±0.3_ _^a^	19.0±0.6_ _^a^	13.0±0.6_ _^a^	14.0±0.6_ _^a^	9.0±0.0_ _^a^	10.3±1.5_ _^a^	10.7±0.9_ _^ad^	14.7±0.9_ _^a^
	780	21.0±0.6_ _^ab^	22.3±0.9_ _^ab^	15.0±2.3_ _^ab^	17.0±0.9_ _^ab^	10.3±0.3_ _^ab^	12.7±0.6_ _^ab^	14.0±0.6_ _^abc^	16.3±2.0_ _^ab^

Gentamicin	0.025	26.0±0.6	28.7±0.6	23.3±0.7	26.3±0.3	21.7±0.6	24.3±0.9	22.7±0.9	25.3±0.3

*M. schimperiana*	195	- - -	7.3±0.3_ _^ad^	- - -	- - -	- - -	- - -	- - -	- - -
	390	8.0±0.6_ _^a^	9.0±1.2_ _^a^	- - -	7.3±0.3_ _^a^	- - -	- - -	- - -	7.3±0.3_ _^a^
	780	11.0±0.6_ _^ab^	12.3±0.3_ _^ab^	8.7±0.9_ _^a^	9.3±0.3_ _^ab^	- - -	- - -	9.0±0.6_ _^ab^	9.7±0.3_ _^ab^

*Trianthema *spp.	195	- - -	7.3±0.3_ _^ad^	- - -	- - -	- - -	- - -	- - -	- - -
	390	- - -	9.7±0.3_ _^a^	- - -	- - -	- - -	- - -	- - -	- - -
	780	9.7±0.7_ _^a^	12.3±0.9_ _^ab^	- - -	- - -	- - -	- - -	- - -	- - -

*S. incanum*	195	- - -	9.7±1.8_ _^ad^	- - -	- - -	- - -	- - -	- - -	- - -
	390	8.7±0.9_ _^a^	13.0±0.9_ _^a^	- - -	7.3±0.3_ _^a^	- - -	7.3±0.3_ _^a^	- - -	7.0±0.0_ _^a^
	780	11±0.7_ _^ab^	15.3±0.9_ _^ab^	- - -	10.3±0.9_ _^ab^	9.3±1.5_ _^ab^	10.0±0.6_ _^ab^	9.0±0.6_ _^ab^	9.7±0.7_ _^ab^

Gentamicin	0.025	26.0±0.6	28.7±0.6	23.3±0.7	26.3±0.3	21.7±0.6	24.3±0.9	22.7±0.9	25.3±0.3

The values are mean ± SEM (n=3); significant at P<0.05; ^a^ compared to gentamicin, ^b^compared to 195mg/ml, ^c^ compared to 390mg/ml, and ^d^compared to 780mg/ml; the negative control has shown no antibacterial activity; Sta.: standard (ATCC) strains; Cli.: clinically isolated strains; - - -: no activity.

**Table 3:** Zone of inhibition (in mm) of combined plant extracts against different bacterial strains.

Plants	Concentration	Bacteria							
		*S. aureus*		*P. aeruginosa*		*E. coli*		*K. pneumoniae*	
	[mg/mL]								
		Cli.	Sta.	Cli.	Sta.	Cli.	Sta.	Cli.	Sta.
CqEh	195	11.3±0.9_ _^ad^	13.0±0.0_ _^ad^	9.7±0.9_ _^acd^	14.0±0.6_ _^acd^	7.7±0.3_ _^ad^	8.7±0.9_ _^ad^	9.3±0.3_ _^ad^	10.7±0.3_ _^ad^
	390	15.0±0.6_ _^a^	16.0±0.6_ _^a^	14.0±0.6_ _^ab^	17.3±0.3_ _^ab^	10.7±0.3_ _^a^	10.7±0.3_ _^ad^	11.7±0.3_ _^ad^	13.0±0.0_ _^a^
	780	18.7±0.3_ _^ab^	19.7±0.3_ _^ab^	17.3±0.9_ _^ab^	19.3±0.9_ _^ab^	13.0±0.0_ _^ab^	14.3±0.9_ _^abc^	15.0±0.6_ _^abc^	15.0±0.6_ _^ab^

CqEp	195	13.0±0.6_ _^ad^	15.0±0.9_ _^ad^	11.0±0.6_ _^acd^	15.3±0.3_ _^ad^	10.7±0.3_ _^ad^	11.0±0.6_ _^ad^	8.7±0.3_ _^acd^	10.7±0.3_ _^acd^
	390	16.3±0.7_ _^a^	18.0±0.6_ _^a^	15.3±0.9_ _^ab^	18.3±0.3_ _^a^	13.3±0.9_ _^a^	14.3±0.3_ _^a^	12.0±0.6_ _^ab^	14.3±0.3_ _^ab^ ^d^
	780	19.3±2.0_ _^ab^	20.7±0.3_ _^ab^	18.0±0.0_ _^ab^	21.3±0.9_ _^ab^	16.3±0.3_ _^ab^	17.0±0.6_ _^ab^	14.3±0.3_ _^ab^	18.0±0.0_ _^abc^

EhEp	195	15.0±0.3_ _^ad^	18.0±0.6_ _^ad^	13.7±0.3_ _^ad^	13.3±0.3_ _^acd^	7.7±0.3_ _^ad^	8.7±0.3_ _^ad^	8.0±0.6_ _^ad^	8.3±0.3_ _^ad^
	390	18.7±0.3_ _^a^	21.7±0.9_ _^a^	16.3±0.3_ _^a^	17.3±0.9_ _^ab^	10.3±0.3_ _^ad^	12.0±0.6_ _^a^	10.3±0.3_ _^a^	10.7±0.3_ _^a^
	780	21.3±0.9_ _^ab^	23.3±0.3_ _^ab^	19.0±0.6_ _^ab^	20.3±0.3_ _^ab^	13.7±0.3_ _^abc^	14.0±0.0_ _^ab^	13.0±0.6_ _^ab^	13.3±1.3_ _^ab^

CqEhEp	195	15.3±0.3_ _^ad^	16.7±0.9_ _^ad^	14.7±0.3_ _^ad^	15.3±0.3_ _^ad^	9.0±0.6_ _^ad^	9.0±0.0_ _^ad^	11.3±0.7_ _^acd^	12.0±0.6_ _^acd^
	390	18.7±0.9_ _^a^	20.3±0.3_ _^ad^	17.7±0.3_ _^a^	18.0±0.6_ _^ad^	11.3±0.3_ _^a^	11.7±0.7_ _^a^	14.3±0.3_ _^abd^	15.7±0.3_ _^ab^
	780	22.3±0.7_ _^b^	24.7±0.9_ _^bc^	21.0±0.0^b^	22.7±0.3_ _^abc^	13.7±0.3_ _^ab^	14.7±0.3_ _^ab^	17.3±0.3_ _^abc^	18.7±0.7_ _^ab^

Gentamicin	0.025	26.0±0.6	28.7±0.6	23.3±0.7	26.3±0.3	21.7±0.6	24.3±0.9	22.7±0.9	25.3±0.3

The values are mean ± SEM (n=3); significant at P<0.05; ^a^ compared to gentamicin, ^b^ compared to 195mg/ml, ^c^ compared to 390mg/ml, and ^d^ compared to 780mg/ml; the negative control has shown no antibacterial activity. Sta.: standard (ATCC) strains; Cli.: clinically isolated strains; - - -: no activity; CqEh:* C. quadrangularis* and *E. heterophylla; *CqEp: *C.quadrangularis* and *E. prostrata*; EhEp: *E. heterophylla *and* E. prostrata*; and CqEhEp = *C. quadrangularis*, *E. heterophylla, *and* E. prostrata*

**Table 4:** The MIC (in mg/mL) of individual studied plant extracts against tested bacteria.

Plants	Bacteria							
	*S. aureus*		*P. aeruginosa*		*E. coli*		*K. pneumoniae*	
	Cli.	Sta.	Cli.	Sta.	Cli.	Sta.	Cli.	Sta.
*C. quadrangularis*	24.38±0.00	24.38±0.00	48.75±0.00	24.38±0.00	390.00±0.00	390.00±0.00	48.75±0.00	48.75±0.00

*C. benghalensis*	24.38±0.00	24.38±0.00	195.00±0.00	97.5±0.00	- - -	- - -	- - -	- - -

*E. heterophylla*	24.38±0.00	24.38±0.00	48.75±0.00	48.75±0.00	48.75±0.00	48.75±0.00	24.38±0.00	24.38±0.00

*E. prostrata*	12.18±0.00	12.18±0.00	24.38±0.00	24.38±0.00	390.00±0.00	195±0.00	48.75±0.00	24.38±0.00

*M. schimperiana*	195.00±0.00	97.5±0.00	- - -	195.00±0.00	- - -	- - -	- - -	195.00±0.00

*S.incanum*	97.5±0.00	48.75±0.00	- - -	195.00±0.00	- - -	390.00±0.00	- - -	390.00±0.00

**Table 5:** The MIC (in mg/mL) of combination of selected studied plant extracts against tested bacteria.

Plants	Bacteria							
	*S. aureus*		*P. aeruginosa*		*E. coli*		*K. pneumoniae*	
	Cli.	Sta.	Cli.	Sta.	Cli.	Sta.	Cli.	Sta.
CqEh	5.08±1.01	5.08±1.01	6.09±0.00	5.08±1.01	24.38±0.00	24.38±0.00	24.38±0.00	24.38±0.00

CqEp	2.54±0.51	2.54±0.51	5.08±1.01	3.05±0.00	6.09±0.00	6.09±0.00	12.18±0.00	6.09±0.00

EhEp	3.05±0.00	2.03±0.88	4.06±1.75	3.05±0.00	12.18±0.00	12.18±0.00	97.5±0.00	48.75±0.00

CqEhEp	3.05±1.01	2.03±0.51	2.54±0.51	2.54±0.51	5.08±1.01	5.08±1.01	6.09±0.00	4.06±1.01

CqEh =*C. quadrangularis *and* E. heterophylla*;* CqEp =C. quadrangularis* and* E. prostrata*; EhEp = *E. heterophylla *and* E. prostrate*; and CqEhEp =*C. quadrangularis, E. heterophylla*,and* E. prostrata*

## Data Availability

Vouchers and dried leaves used for this study are stored at the Herbarium of the Department of Plant Science, Haramaya University, Ethiopia. The datasets supporting the conclusion of this study are available from the corresponding author on reasonable request.

## Abbreviations

ATCC: American Type Culture Collection
CLSI: Clinical and Laboratory Standard Institute;
CqEh: * C. quadrangularis and E. heterophylla*
CqEhEp: * C.quadrangularis, E. heterophylla, and E. prostrate*
CqEp: * C. quadrangularis and E. prostrata*
EhEp: * E. heterophylla and E. prostrate*
MIC: Minimum inhibitory concentration
MHA: Muller-Hinton agar.
